# Correspondence between the Graduating Class of the Baltimore College and Dr. A. Westcott

**Published:** 1847-03

**Authors:** 


**The following correspondence should have been published along with the valedictory address of Professor Westcott, but was inadvertently omitted:**

**College Buildings,**
***Baltimore, Feb'y 16th,* 1847.**

**Prof. A. Westcott,**

**In consideration of the ability and excellence which characterised your Valedictory Address to the Graduating Class of the Baltimore College of Dental Surgery, beside the high respect we entertain towards you personally, we do, most respectfully, request a copy of the same for publication—also that it be published in the American Journal of Dental Science, together with the correspondence. By a compliance with this request, you will confer a favor upon**

JOHN C. BAGBY,
JAS. W. McCULLOH,
J. N. BAIRD,
JOHN D. WEMPLE,
THOS. PALMER,
} *Graduating Class.*

**Baltimore, *Feb'y 16th,* 1847.**

***To the Graduating Class of the Baltimore College of Dental Surgery:***

**Gentlemen—Your flattering request has just been placed in my hands. In reply, allow me to offer you my most sincere acknowledgments for the high compliment you bestow upon me, and to give you the assurance, that from no source could it be more gratifying. I herewith transmit the manuscript of my address, which is at your disposal.**

**With sentiments of the highest esteem and respect,**

**I am, most cordially,**

**Your friend and ob't serv't,**

**A. WESTCOTT.**

**Messrs. Bagby, Wemple and others.**

